# Molecular Viability Testing of Bacterial Pathogens from a Complex Human Sample Matrix

**DOI:** 10.1371/journal.pone.0054886

**Published:** 2013-01-24

**Authors:** Kris M. Weigel, Kelly L. Jones, Julie S. Do, Jody Melton Witt, Jae-Hyun Chung, Christian Valcke, Gerard A. Cangelosi

**Affiliations:** 1 Department of Environmental and Occupational Health Sciences, University of Washington, Seattle, Washington, United States of America; 2 AttoDx, Inc., Seattle, Washington, United States of America; 3 Novartis Diagnostics, Emeryville, California, United States of America; 4 Department of Mechanical Engineering, University of Washington, Seattle, Washington, United States of America; University of Padova Medical School, Italy

## Abstract

Assays for bacterial ribosomal RNA precursors (pre-rRNA) have been shown to distinguish viable from inactivated bacterial cells in drinking water samples. Because the synthesis of pre-rRNA is rapidly induced by nutritional stimulation, viable bacteria can be distinguished from inactivated cells and free nucleic acids by measuring the production of species-specific pre-rRNA in samples that have been briefly stimulated with nutrients. Here, pre-rRNA analysis was applied to bacteria from serum, a human sample matrix. In contrast to drinking water, serum is rich in nutrients that might be expected to mask the effects of nutritional stimulation. Reverse transcriptase quantitative polymerase chain reaction (RT-qPCR) assays were used to detect pre-rRNA of four bacterial species: *Acinetobacter baumannii, Pseudomonas aeruginosa, Staphylococcus aureus,* and the *Mycobacterium tuberculosis* complex. These species were chosen for their clinical significance and phylogenetic diversity (Proteobacteria, Firmicutes, and Actinobacteria). To maximize resolving power, pre-rRNA was normalized to genomic DNA of each pathogen. When viable cells were shifted from serum to bacteriological culture medium, rapid replenishment of pre-rRNA was always observed. Cells of *P. aeruginosa* that were inactivated in the presence of serum exhibited no pre-rRNA response to nutritional stimulation, despite strong genomic DNA signals in these samples. When semi-automated methods were used, pre-rRNA analysis detected viable *A. baumannii* cells in serum at densities of ≤100 CFU/mL in <5.5 hours. Originally developed for rapid microbiological analysis of drinking water, ratiometric pre-rRNA analysis can also assess the viability of bacterial cells derived from human specimens, without requiring bacteriological culture.

## Introduction

Bacterial pathogens such as *Acinetobacter baumannii*, *Pseudomonas aeruginosa*, *Staphylococcus aureus,* and *Mycobacterium* species, are common causes of healthcare-associated infections. Laboratory diagnosis of such infections must balance the needs of sensitivity, speed, and clinical relevance. Gold standard bacteriological culture is sensitive and specific to viable pathogen cells; however the time required (from 1 to 30 days depending on species) is problematic in the context of life-threatening infections. Moreover, supplementary testing is needed to identify pathogen species.

The polymerase chain reaction (PCR) is a fast, sensitive, and specific alternative to culture [1], [2]. However, PCR cannot distinguish viable bacterial cells from non-viable cells, or from free nucleic acids (NA) in samples. Because RNA is less stable than DNA, tests for ribosomal RNA (rRNA) or messenger RNA (mRNA) have been described to improve detection of viable pathogens [3]–[10]. However, bacterial mRNA is unstable and difficult to detect [11], while rRNA can persist in dead bacterial cells [1]. Another approach involves treating bacteria with DNA intercalators that penetrate inactivated cells and inhibit PCR amplification, but are excluded from viable cells [12], [13]. These methods are effective but performance varies with sample conditions [14]–[17].

In order to improve specificity for viable cells, we have developed assays for bacterial rRNA precursors (pre-rRNA) [18]. Pre-rRNAs are intermediates in rRNA synthesis generated by rapid nucleolytic cleavage of *rrs-rrl-rrf* operon transcripts. Leader and tail fragments are subsequently removed to yield mature rRNA. In growing bacterial cells, pre-rRNAs are more abundant and easier to detect than the most strongly-expressed mRNAs. Pre-rRNAs were estimated to account for 25% of total rRNA in growing *Acinetobacter* cells [19]. Therefore, the copy number of pre-rRNA in growing cells may exceed that of all mRNA molecules combined. Moreover, pre-rRNAs have species-specific sequences that facilitate species identification in complex samples. When bacterial growth slows, pre-rRNA synthesis declines but its processing continues, resulting in active drainage of pre-rRNA pools [20]. Pre-rRNA is rapidly replenished when growth-limited cells are given fresh nutrients [20]–[22]. Such fluctuations occur consistently in viable cells but are not seen in dead cells or with free nucleic acids. Mature rRNA can exhibit similar nutrition-dependent fluctuations, but the magnitudes of such fluctuations are far exceeded by those of pre-rRNA [19], [20], [23], [24].

In a previous study we reported a pre-rRNA-targeted RT-qPCR test that detected viable cells of the enteric pathogen *Aeromonas hydrophila* in tap and surface water samples [18]. To conduct the test, samples were split into two aliquots, one of which was nutritionally stimulated. When viable *A. hydrophila* cells were present, pre-rRNA increased in the stimulated aliquot relative to the non-stimulated aliquot. Pre-rRNA stimulation was very rapid in viable cells (<1 generation time). Non-viable cells did not exhibit this increase. This strategy was termed ratiometric pre-rRNA analysis.

In the present study, pre-rRNA analysis was applied to bacteria derived from a biological matrix, human serum. In contrast to water, serum is rich in nutrients that could enable bacterial replication and the maintenance of large pre-rRNA pools, thus diminishing the resolving power of ratiometric pre-rRNA analysis. However, the balanced nutritional conditions of laboratory media are rare in nature, where microbial growth is nearly always limited by the availability of specific nutrients. Therefore, we hypothesized that the provision of limiting nutrients will stimulate pre-rRNA synthesis in bacteria derived from biological samples.

The four bacterial species examined in this study–*Acinetobacter baumannii, Pseudomonas aeruginosa, Staphylococcus aureus*, and the *Mycobacterium tuberculosis* complex (MTBC)–were chosen for their phylogenetic diversity as well as for their clinical significance. Enzymatic pathways of rRNA maturation differ between Gram-negative and Gram-positive bacteria [22], [25]–[27]. Whereas pre-rRNA pools have been well characterized in Gram-negative bacteria [18]–[20], [23], [28], the same is less true of Gram-positive bacteria and Actinobacteria. The present study also introduced refinements that improved the resolving power and throughput of pre-rRNA analysis. This is the first report of ratiometric pre-rRNA analysis conducted on bacteria present in a human sample matrix.

## Materials and Methods

### Nutritional Stimulation Time Course and Pre-rRNA Analysis

Most experiments assessed pre-rRNA levels over a time course of nutritional stimulation, These experiments were performed as follows. Cells of *A. baumannii* (ATCC 17978), *P. aeruginosa (*ATCC BAA-47, strain HER-1018/PAO1), *S. aureus* (ISP 479-), and MTBC (*M. bovis* BCG [Russia] and *M. tuberculosis* H37Ra) were grown at 37°C to early stationary-phase in 10 mL broth in 50 mL polypropylene conical tubes, shaking at 50–100 rpm. *M. bovis* BCG and *M. tuberculosis* H37Ra were grown in Middlebrook 7H9 broth supplemented with 10% ADC (VWR) and 0.05% Tween 20, while the other three organisms were grown in trypticase soy broth (TSB). Cells were centrifuged at 16000×g in 1.5 mL tubes for two minutes, washed once with 1 mL PBS, pH 7.4, and resuspended in 10 or 25 mL human serum, type A positive (heat inactivated at 56°C for 45 min by the supplier, Interstate Blood Bank, Inc.) at final densities of approximately 1E8 CFU/mL (estimated by turbidity). Suspensions in serum were incubated for 7 days (MTBC for 30 days) in 250 mL baffled flasks with moderate shaking at 37°C.

Prior to nutritional stimulation of the fast-growing species (*A. baumannii, P. aeruginosa,* and *S. aureus*), control (non-stimulated) samples were collected by centrifuging 50 µL aliquots of the serum cell suspensions. Pellets were aspirated and stored at −80°C until DNA and RNA analysis. In addition, serial dilutions of the suspensions were plated on trypticase soy agar (TSA) for CFU enumeration. To initiate nutritional stimulation, serum-acclimated cultures were diluted 1∶10 in fresh TSB by adding 2.5 mL aliquots of each serum cell suspension directly to 22.5 mL pre-warmed TSB in a 250 mL baffled glass flask. The flask was incubated with shaking at 37°C. At various time points following the initiation of nutritional stimulation, 500 µL samples were withdrawn and centrifuged. These samples were 10-fold greater in volume than the stored non-stimulated samples in order to compensate for the 10-fold dilution into TSB. Stimulated cell pellets were stored at −80°C until DNA and RNA measurement, thereby ensuring that both stimulated and non-stimulated aliquots were handled and frozen similarly.

Nutritional stimulation of slow-growing MTBC cells was performed similarly, with the following modifications: Pre- and post-enrichment samples were 0.5 mL and 5 mL respectively, CFU enumeration was on supplemented Middlebrook 7H10 agar, and nutritional enrichment was performed in supplemented Middlebrook 7H9 broth.

DNA and RNA (TNA) were simultaneously extracted from frozen cell pellets as described previously [18]. Briefly, cells were lysed by bead beating in sodium acetate-sodium dodecyl sulfate-EDTA lysis buffer and acidified phenol. Cooled lysates were centrifuged and supernatants washed with chloroform-isoamyl alcohol (24∶1) before the TNA was cold-precipitated in acidified isopropanol. The precipitate was washed in 75% ethanol, dried, and resuspended in 100 µL DEPC-treated deionized water, of which 10 µL was retained for DNA quantification by qPCR. Pre-rRNA was measured in the remaining 90 µL.

**Figure 1:**
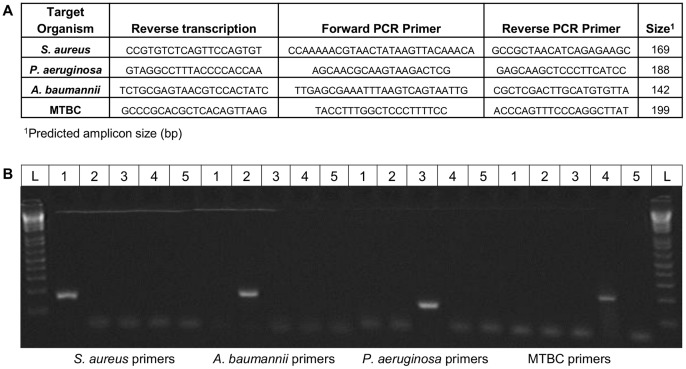
Reverse transcription and PCR primers. A: Oligonucleotides used for priming reverse transcription of pre-rRNA (cDNA synthesis), and forward and reverse PCR primers for amplifying cDNA. B: Specificity of PCR primer sets designed for *S. aureus, A. baumannii, P. aeruginosa*, and MTBC. Each primer set was used in endpoint PCR conducted on purified DNA from all the four organisms. Key: L, 1 Kb+ DNA ladder; 1, *S. aureus* DNA; 2, *A. baumannii* DNA; 3, *P. aeruginosa* DNA; 4, *M. tuberculosis* DNA; 5, negative control (water). Lowest three bands of ladder are 100, 200, and 300 bp. A repeat experiment yielded identical results.

For pre-rRNA measurement, complementary DNA (cDNA) was generated following a strategy described previously [18]. Briefly, the resuspended TNA was cleaned (Qiagen RNeasy kit, 74104) and up to 4 µg TNA was mixed with 0.4 µM (final concentration) gene-specific oligonucleotide primer in 10 µL buffer. The primer was complementary to a region downstream of the 5′ terminus of the mature 16S rRNA of each species, and designed to prime reverse transcription toward the pre-16S leader region known as the external transcribed spacer (ETS1) (Fig. 1A). The mixture (RNA and primers) was denatured at 65°C for 5 minutes, rapidly cooled on wet ice for 2–5 minutes, and supplemented with 4 µL 5X First Strand buffer, 2 µL 0.1 M DTT, 1 µL 10 mM dNTPs, 2 µL DEPC-treated H_2_O, and 1 µL SuperScript III RT enzyme (Invitrogen Cat# 18080-044). These reactions were incubated at 50°C for 50 minutes. The remaining RNA was hydrolyzed with NaOH, and the more stable cDNA cleaned using Qiagen’s PCR purification kit and eluted in 50 µL elution buffer.

Quantitative PCR (qPCR) amplification of cDNA or genomic DNA (gDNA) was run on an ABI Prism FAST RT-7500 PCR system using the Applied Biosystems Power SYBR Green mix (4367659). Samples were run in triplicate along a range of appropriate dilutions; briefly, 2 µL templates were added to 0.25 µL (7.5 nmoles) of each primer, 10 µL 2×SYBR mix, and 7.5 µL H2O to a final volume of 20 µL per well. The primer sets bridged the earlier-described junction between ETS1 and the mature 16S rRNA (Figure 1). Standard curves were generated using 10-fold serially-diluted genomic DNA (gDNA) for quantification of pre-rRNA and gDNA amplified by using the same primers. The gDNA used for standard curves was extracted from bacterial cultures by using commercial DNA purification kits. Its quantity (in ug/mL) and purity was assessed spectrophotometrically. Reaction conditions were as follows: 10 minutes 95°C, 40 cycles of (15 s at 95°C, 30 s at 57°C, 30 s at 72°C) using ‘9600 emulation.’ ABI’s software (7500 Fast System SDS v1.4.025, and v2.0.5) was used to automatically set Ct threshold values, except when manual adjustment into the exponential range was necessary. The results were exported to a spreadsheet for analysis. From separate gDNA standard curves in each experiment, qPCR efficiencies were calculated [10^(−1/slope)^ −1].

To calculate ratios of pre-rRNA to gDNA (P:G) in nutritionally stimulated and non-stimulated samples, inputted pre-rRNA and gDNA were quantified relative to a gDNA standard curve amplified with the same primers. Standard deviations were calculated from all possible ratiometric permutations.

### Serum Acclimation Time Course

In a second set of experiments, human serum was inoculated with a smaller number of cells (∼1E5/mL) and acclimated in serum for shorter periods (as little as four hours). *A. baumannii*, *P. aeruginosa*, and *S. aureus* were cultured overnight as above. Cells were removed, washed and resuspended in PBS for OD_600_ measurement. Triplicate 5 mL pre-warmed serum suspensions of each organism were prepared at ∼1×10^5^ CFU/mL in 14 mL round-bottom tubes and incubated shaking at 37°C. After acclimation in serum for 4, 24, and 168 hours, sample collection and nutritional stimulation were initiated as above with the following modifications: unstimulated samples were 250 µL and stimulated samples were created by adding 250 µL serum suspension to 2.5 mL pre-warmed TSB followed by a 1.5 hour incubation in 14 mL round bottom tubes. In both cases, the entire aliquot was pelleted and washed 3× in PBS before storage at -80°C. Initial inoculum densities and viability at each serum incubation timepoint were assesed by serial CFU plating of each replicate.

DNA and RNA were simultaneously extracted using Epicentre’s MasterPure Complete DNA and RNA purification kit. Where necessary, total cell pellet size was reduced by dilution to ≤5E8 CFU before extraction. The furnished protocols were followed with the following changes. Cell pellets were resuspended and pre-incubated in 100 µL TE containing lysozyme and/or lysostaphin. *A. baumannii* and *P. aeruginosa* were pre-lysed for 5 minutes in TE with 0.5 µg/µL lysozyme at 37°C; *S. aureus* was pre-lysed for 15 minutes in TE with 5 µg/µL lysozyme and 0.25 µg/µL lysostaphin at 37°C. Then 200 µL 2× lysis buffer (supplied) with 1 µg/µL proteinase K was added to each sample and incubated at 65°C for 15 minutes (*A. baumannii and P. aeruginosa*) or 30 minutes (*S. aureus*). TNA was eluted in 25 µL TE, from which 5 µL was removed for DNA measurement. From the remaining 20 µL, RNA was purified by DNase I treatment and re-precipitation, as directed by the included protocol. Purified RNA was eluted in 20 µL TE and characterized spectrophotmetrically.

For pre-rRNA measurement, cDNA was created as above, but with Promega’s ImProm-II Reverse Transcription system. Up to 500 ng RNA was reverse-transcribed in each 20 µL reaction containing 3 mM MgCl_2_, with RNase-inhibitor added before denaturation.

Quantification of pre-rRNA and gDNA by qPCR was peformed as described above. To express changes in pre-rRNA levels following nutritional stimulation, each replicate’s stimulated P:G ratio was divided by its unstimulated P:G ratio (P:G+/P:G-). Means and standard deviations of these values were calculated for each timepoint and organism. Samples with viable cells are expected to generate ratios <1.

### Semi-automated Pre-rRNA Measurement

*A. baumanii* strain ATCC 17978 was cultured overnight as described above. A 50 µL aliquot was transferred to 3 mL of human serum (PAA Laboratories, C11-021) and incubated in a 37°C rotary shaker at 225 rpm. After five days, the serum was diluted 2E5, 1E6, 2E6, 1E7, 2E7, and 1E8-fold in fresh serum. In addition, a sample of the undiluted serum was serially diluted and plated onto TSA containing 5% sheep blood for CFU enumeration. To conduct ratiometric pre-rRNA analysis, a 100 µL sample of each dilution was immediately added in duplicate to 900 µL PBS (non-stimulated control) or to 900 µL pre-warmed TSB (stimulated samples). Non-stimulated samples were centrifuged, supernatant was removed, and pellets were stored at −80°C for RNA extraction at a later time. Stimulated samples were incubated for 90 minutes in a 37°C rotary shaker at 225 rpm and were frozen and stored as described above.

Nucleic acid was extracted from frozen bacterial pellets by using the Blood Cell Storage Inc. (BCSI) Nucleic Acid Extraction System [29]. Pellets were briefly thawed at room temperature, and 380 µL of TE plus 1% SDS (lysis buffer) and 10 µL of 20 mg/mL Proteinase K (VWR IB05406) were added to each pellet, vortexed for 20 seconds, and placed in a 60°C incubator for 10 minutes. Then, 400 µL each of GT Lysis Buffer and >95% ethanol were added to the tube with brief vortexing after the addition of each reagent. The entire volume was loaded onto a nucleic acid extraction (NAE) card following kit instructions and incubated at room temperature for 15 minutes. The cards were loaded onto the NAE automated system with the following wash parameters: 3×1 mL Wash 1; 3×1 mL Wash 2; and 4 minutes of drying time. Cards were manually eluted with 100 µL elution buffer (TE).

One-step reverse transcription and qPCR amplification was performed on the Applied Biosystems 7500 Fast Real-Time PCR system using the Thermo Verso 1-step QRT-PCR kit (AB-4100/C). Cycling parameters were as follows: 50°C for 30 minutes, 95°C for 15 minutes, and 45 cycles of 95°C for 15 seconds and 60°C for 1 minute. For each 25 µL reaction, suggested volumes were used for the Verso Enzyme Mix and the 1-Step QPCR Mix. Final concentrations were 400 nM for primers, 200 nM for probe, and 200 nM for the ROX passive reference dye, with 5 µL of each extraction template added. Primers and probe were ordered from Eurofins MWG Operon: Abaum-RPRA-F1 and Abaum-RPRA-R1 (see Figure 1), and probe [6∼FAM] CTG CCG CCA GCG TTC AAT CTG AGC CAT G [TAMRA∼6∼FAM]. Increases in pre-rRNA in nutritionally stimulated aliquots relative to non-stmulated (PBS) controls are expressed as the difference in Ct value between stimulated and non-stimulated aliquots (ΔCt).

## Results

### Specificity of RT-qPCR Assays for Pre-rRNA

In all four bacterial species, RT-qPCR reactions detected the 5′ ETS1 region upstream of the small subunit (SSU) rRNA-encoding gene. Primer sets were designed to straddle the 5′ mature rRNA terminus as described previously [18]. Primers for cDNA synthesis and reverse qPCR primers recognized semi-conserved sequences within the mature rRNA (16S), while forward primers recognized species-specific sequences within the ETS1. Therefore, successful amplification required intact pre-rRNA molecules (or gDNA) as templates.

Figure 1A shows the primer sequences used for both manual and semi-automated pre-rRNA measurements. When possible, reverse transcription and reverse PCR primers targeted mature SSU rRNA sequences with at least some phylogenetic specificity; however, each assay primarily derived its specificity from the forward primer targeting the hypervariable ETS1. All forward primers were predicted to be species-specific based on BLAST searches conducted against the NCBI non-redundant database. Consistent with this prediction, when PCR primer sets for *A. baumannii, P. aeruginosa, S. aureus,* and MTBC were applied to purified DNA from all four species, no cross-reactivity was observed in end-point PCR (Figure 1B) or qPCR.

### Time Courses of Nutritional Stimulation of Rapidly-growing Species after Serum Acclimation

Given the abundance of carbon and energy sources in serum, we asked whether bacteria that had been incubated in serum exhibit rapid pre-rRNA synthesis in response to nutritional stimulation. We also used traditional plating to determine the viability of cells incubated in serum. Cells of rapidly-growing Gram-positive (*S. aureus*) and Gram-negative (*P. aeruginosa*, *A. baumannii*) bacteria were incubated in serum for time periods ranging from 4 hours to 7 days at 37°C, then plated onto TSA to determine viability. The strains used in this analysis differed in their tolerance to two lots of serum used in this study. The first lot (ZX049642) was used in two replicate experiments in which bacteria were incubated in serum for 5–7 days, followed by analysis of the effects of nutritional stimulation for periods ranging from 1–4 hours. The second lot of serum (BX053838) was used in separate experiments in which we performed a serum acclimation time course, incubating the bacteria for 4, 24, and 168 hours in serum followed by a 90 minute nutritional stimulation.

The *A. baumannii* strain thrived in the first lot of serum, increasing in numbers from an estimated 1E8 CFU/mL on day 0 to 9E8 CFU/mL on day 7 (and to 2.9E9 in a replicate experiment). In the second lot, it fared less well–after four hours in serum, the initial 1.2E5 CFU/mL were reduced to 5E3 CFU/mL before eventually recovering to 8.6E4 CFU/mL by day seven. Viable counts of *S. aureus* decreased from an estimated 1E8 to 4E4 CFU/mL over 7 days in the first serum lot (9.7E5 in the replicate experiment). In the second lot the viability was relatively unchanged from the initial density of 6.4E4 CFU/mL, peaking at 4E5 CFU/mL after 4 hours before declining to 3E4 CFU/mL by 7 days. The *P. aeruginosa* isolate exhibited radically different tolerance to the two lots of serum. It lost all detectable viability after 7 days incubation in the first lot, decreasing from an estimated 1E8 to <1E2 CFU/mL in both experiments. However, it grew throughout the time course in the second lot, increasing from 1.1E5/mL to 5.4E9/mL by day seven.

The time courses of nutritional stimulation on pre-rRNA pools in serum-acclimated cells were evaluated. Each cell suspension in serum was divided into two aliquots and centrifuged. One pellet was retained as a non-stimulated (“0-hour”) control aliquot while the other was resuspended in TSB and incubated at 37°C for up to 4 hours. Samples were taken after 1, 2, and 4 hours of nutritional stimulation. Pre-rRNA and genomic DNA were quantified by qPCR with a common primer set. By normalizing pre-rRNA to genomic DNA in all samples, cellular pre-rRNA abundance could be compared between samples, with minimal effects from variations in nucleic acid extraction efficiency or the presence of PCR inhibitors. Ratios of pre-rRNA to gDNA (P:G) in stimulated and control samples were calculated from the results. Because of the inefficiency of RT-qPCR relative to qPCR, P:G ratios usually appeared to be 1 or less. In reality, pre-rRNA copies per genome number in the hundreds or more, as indicated by past experiments in which pre-rRNA and gDNA were detected by direct probe analyses [20].

**Figure 2:**
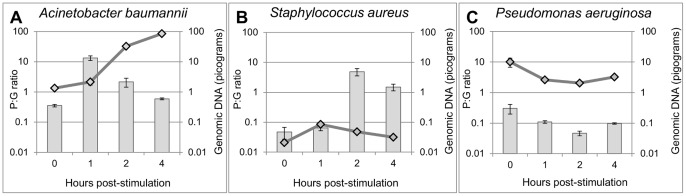
Ratiometric pre-rRNA analysis of *A. baumannii*, *S. aureus*, and *P. aeruginosa* cells in serum. A–C: Analysis of cells that had been held in serum for 7 days. Nutritional stimulation was initiated by suspending cells in pre-warmed TSB, and samples taken after 0, 1, 2, and 4 hours were subjected to RT-qPCR and qPCR to quantify pre-rRNA and gDNA, respectively. The same primers were used to amplify gDNA and cDNA generated from pre-rRNA. Ratios of pre-rRNA to gDNA (P:G; bars) are means and SDs of nine ratiometric permutations from three technical replicates of each sample type. Quantity of gDNA (lines) are means and standard deviations of the three gDNA measurements. Viable cell densities of *A. baumannii, S. aureus,* and *P. aeruginosa,* respectively, in serum were 9.0×10^8^, 9.7×10^5^, and <1×10^2^ CFU/mL. From separate gDNA standard curves consisting of ≥ five points each, qPCR efficiencies were calculated [10^(−1/slope)^ −1] to be between 0.913 and 0.959. A replicate experiment (Figure S1) yielded similar results for all three organisms.

Pre-rRNA stimulation correlated with viability in serum-derived bacteria. *A. baumannii,* which survived and grew in serum as determined by CFU plating, exhibited robust increases in the P:G ratio within 1 hour of nutritional stimulation (bars in Figure 2A). During this 1-hour period genomic DNA increased marginally if at all (line in Figure 2A). The P:G ratio began to decline after 2 hours post-stimulation, at which point the cells had already initiated active replication as indicated by genomic DNA signals. Relative to *A. baumannii,* the P:G ratio in *S. aureus* increased more slowly, peaking at 2 hours, with genomic DNA signals remaining low throughout the experiment (Figure 2B). *S. aureus* cells may have lysed in the serum, leaving behind a small number of intact cells. Some of these cells were viable as indicated by the plating results, and sufficient to yield robust pre-rRNA increases after nutritional stimulation.

In contrast to *A. baumannii* and *S. aureus, P. aeruginosa* cells did not survive incubation in this lot of serum, as determined by plating. These cells exhibited no detectable pre-rRNA synthesis upon nutritional stimulation. Genomic DNA signals were about as high in *P. aeruginosa* as they were in *A. baumannii,* indicating the persistence of intact cells and/or cell-free *P. aeruginosa* DNA (Figure 2C). The observation that serum-inactivated *P. aeruginosa* cells do not synthesize pre-rRNA upon nutritional stimulation was consistent with previous observations made on hypochlorite-inactivated *A. hydrophila* cells in water [18]. Viable *P. aeruginosa* cells derived from the second lot of serum exhibited robust pre-rRNA production upon nutritional stimulation (below); therefore, the lack of pre-rRNA production in spiked serum was not due to a unique inability of *P. aeruginosa* cells to produce pre-rRNA in response to nutritional stimulation.

These observations illustrate the utility of ratiometric pre-rRNA analysis. The ‘0’- hour (pre-stimulation) measurements of gDNA in Figure 2 resemble standard PCR-based diagnostic tests, in that material taken directly from samples was subjected to PCR to detect the DNA of a targeted species, regardless of viability. These would have appeared strongly positive for *A. baumannii* and *P. aeruginosa,* and more weakly positive for *S. aureus.* Such findings would have been inconsistent with plating results, which showed that few if any viable *P. aeruginosa* survived incubation in serum. Ratiometric pre-rRNA results were more consistent with plating, with *P. aeruginosa* yielding a negative result and the other two species yielding positive results by this method.

### Serum Acclimation Time Courses

In order to determine whether the results in Figure 2 depended on high cell densities and/or extended acclimation to serum, a second set of experiments was conducted in which a second lot of human serum was inoculated with a smaller number of cells (∼1E5/mL) and acclimated in serum for shorter periods (as little as four hours). In these experiments viable *A. baumannii*, *P. aeruginosa,* or *S. aureus* cells were present in each replicate at every time point tested (4, 24, and 168 hours), according to plating results. It is not known whether the improved survival of *P. aeruginosa* in this experiment was due to differences in seeding density, serum lot, or both. In agreement with plating results, pre-rRNA production–revealed by increased P:G ratios in stimulated samples relative to unstimulated samples–indicated viable cells were present in all replicates tested.

**Figure 3:**
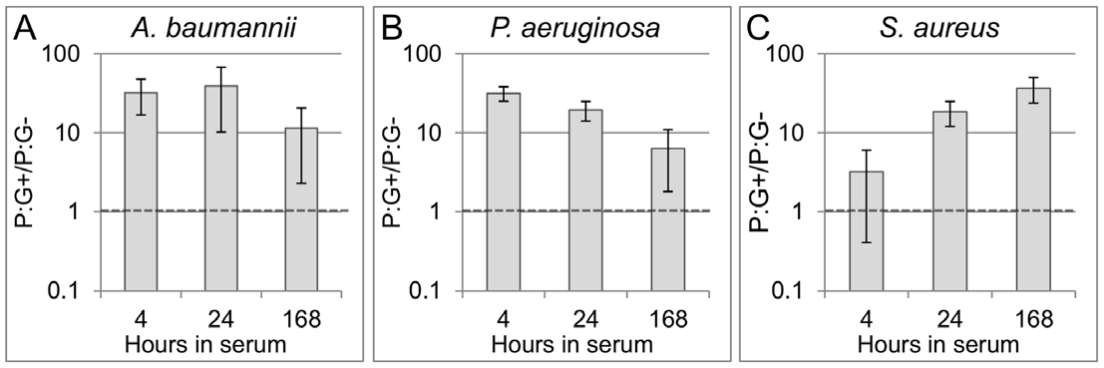
Ratiometric pre-rRNA analysis of *A. baumannii* (A), *P. aeruginosa* (B), and *S. aureus* (C) cells in serum over time. Three biological replicates for each organism were prepared at ∼1E5 CFU/mL in serum and analyzed after 4, 24, and 168 hours of serum acclimation. At each timepoint, nutritional stimulation was initiated by suspending cells in pre-warmed TSB for 1.5 hours. Changes in pre-rRNA are expressed as means and standard deviations of the fold-increases in P:G ratio following nutritional stimulation, relative to non-stimulated control aliquots (P:G+/P:G−). The horizontal dashed line indicates the “viability threshold” which samples with viable cells are expected to exceed. From separate gDNA standard curves consisting of ≥ five points each, qPCR efficiencies were between 1.010 and1.067.

For *A. baumannii* and *P. aeruginosa*, nutritional stimulation strongly induced pre-rRNA production in each biological replicate, increasing P:G ratios >10-fold, after only four hours in serum (Figure 3A and 3B). After seven days (168 hours) in serum, nutritional stimulation induced pre-rRNA synthesis that was less robust in both organisms, although still significant (P:G+/P:G−>1) for each replicate. *S. aureus* appeared to require longer incubation in serum to thoroughly acclimate and drain its pre-rRNA pools (Figure 3C). However, 24 hours of acclimation to serum resulted in sufficient drainage of pre-rRNA pools to yeild a >10-fold increase in pre-rRNA upon nutritional stimulation. Thus, the results in Figure 2 did not depend on high cell density and/or extended (7 days) acclimation to serum.

### Pre-rRNA Analysis of a Slow-growing *Mycobacterium* Species

**Figure 4:**
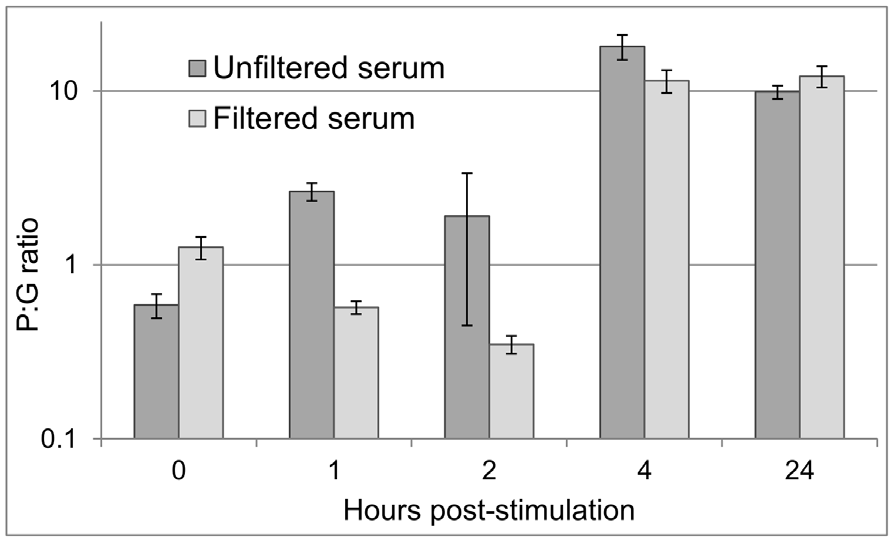
Ratiometric pre-rRNA analysis *M. bovis* BCG cells in serum. Cells were incubated separately in filtered and unfiltered human serum at 37°C for 30 days. The serum-incubated cells were then resuspended in pre-warmed 7H9 broth and samples were taken after 0,1, 2, 4, and 24 hours. P:G ratios were calculated as in Figure 2. Values are means and SDs of nine ratiometric permutations from three technical replicates of each sample type. From a five-point gDNA standard curve, qPCR efficiency was calculated as 1.001. A separate experiment with H37Ra yielded similar results (Figure S2).

The three species in Figures 2 and 3 share the physiological property of rapid aerobic growth. With the requisite viability, all exhibited rapid pre-rRNA upshift (1–2 generation times) in response to nutritional stimulation after incubation in sub-optimal conditions. To determine whether the same is true of slow-growing bacterial species, we examined *M. bovis* BCG cells that had been incubated in filtered or unfiltered human serum for 30 days at 37°C. When transferred to supplemented Middlebrook 7H9 broth, these cells exhibited dramatic pre-rRNA upshift in 1 to 4 hours, a fraction of their normal 24 hour generation time (Figure 4). Similar results were obtained with a related strain, *M. tuberculosis* H37Ra (Figure S2). Separate plating experiments indicated that both species survive serum exposure well and were viable after 30 days (data not shown). Thus, slow-growing mycobacteria in serum respond to nutritional stimulation in a similar fashion to fast-growing Gram-negative and Gram-positive bacteria.

### Semi-automated Pre-rRNA Analysis

The preceding results demonstrate the biological feasibility of molecular viability testing in a complex human sample matrix. However, these samples were spiked to high cell densities (≥1E5 CFU/mL). In addition, the experiments used labor-intensive manual methods described previously [18].

**Figure 5:**
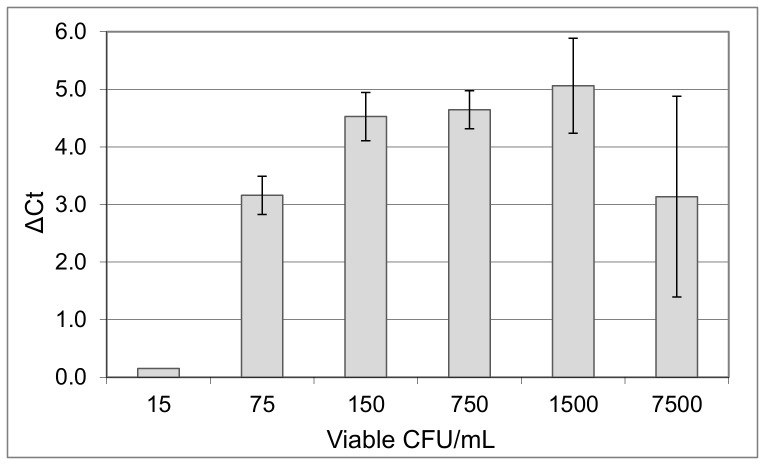
Ratiometric pre-rRNA analysis of *A. baumannii* cells in serum by using a rapid semi-automated approach. Serum-incubated cells were plated to quantify viable CFU/mL, then serially diluted in serum. Viable CFU/mL values (X-axis) were subsequently calculated from plating results. To initiate nutritional stimulation, dilutions in serum were further diluted 1∶10 into TSB or PBS (stimulated and non-stimulated, respectively). After 90 minutes, pre-rRNA was quantified by one-step probe-based q-RT-PCR as described in text. Values are means and SDs of ΔCt values (non-stimulated minus stimulated) from two replicates of each dilution. Control samples with no bacteria (0 CFU/mL) yielded no RT-qPCR results, and therefore could not be plotted as ΔCt values. Reaction efficiency could not be calculated for this experiment, because no standard curve was used. A replicate experiment (Figure S3) yielded similar results, showing a limit of detection of ≤56 viable CFU/mL.

To better evaluate the practical feasibility of ratiometric pre-rRNA analysis as a diagnostic strategy, a more streamlined semi-automated approach was applied to serum samples with spiked *A. baumannii* cells present at lower viable cell densities ranging from 15 to 7500 CFU/mL, as determined by viability plating. The detection protocol used automated nucleic acid extraction cards [29] and the single-step RT-qPCR method described in Materials and Methods. It required 5.5 hours to complete, including a 1.5-hour nutritional stimulation. DNA was not quantified and ratios of pre-rRNA to gDNA (P:G) were not calculated. Instead, pre-rRNA in nutritionally stimulated samples was quantified relative to pre-rRNA in non-stimulated (time 0) controls. The results were expressed as the difference in Ct values between stimulated and non-stimulated aliquots (ΔCt). Here, ΔCt values did not consistently increase with increasing viable cell numbers, because the proportions of viable to inactivated cells were roughly similar in each sample. In four experiments conducted as shown in Figure 5, positive ΔCt values were always observed when viable cell densities were ∼100 CFU/mL serum or more (a typical replicate is shown in Figure S3). Therefore, ratiometric pre-rRNA analysis can be applied to samples with physiologically relevant cell densities, in a procedure that yields results faster than bacteriological culture.

## Discussion

This study asked whether ratiometric pre-rRNA analysis, which was originally developed for analysis of drinking water, could also assess the viability of bacterial cells in human specimens. Although human fluids such as serum are rich in certain nutrients, they are usually limited in some required nutrients, and bacterial growth rates are sub-maximal in such environments. Therefore, we hypothesized that the provision of limiting nutrients would stimulate pre-rRNA synthesis in viable bacteria derived from such samples. The results showed this to be true for all organisms tested. When bacteria were not viable, as seen in *P. aeruginosa* after incubation in the first lot of serum, nutritional stimulation failed to elicit the production of pre-rRNA.

The five species used in this study were phylogenetically diverse, with the bacterial phyla Proteobacteria, Firmicutes, and Actinobacteria represented. They were also diverse physiologically, with laboratory generation times ranging from <1 hour to ≥24 hours and varying numbers of rRNA biosynthetic genes. All of them responded to nutritional stimulation in similar fashion, producing significant amounts of pre-rRNA in less than 1–2 generation times, even after extended incubation in growth-limiting conditions. *S. aureus* sometimes exhibited a delayed response to changing nutritional environments. Relative to the other two species, it required longer periods to drain pre-rRNA pools in serum, and its response to nutritional stimulation peaked at 2 hours rather than 1 hour. These observations might be explained by its relatively slow growth rate. Despite these variations, we have found that a 90 minute nutritional stimulation consistently induces a strong pre-rRNA signal in *A. baumannii, P. aeruginosa,* and *S. aureus.* Pre-rRNA synthesis is among the earliest steps in growth initiation, significantly outpacing DNA replication and cell division. The phylogenetic conservation of this phenomenon enhances the utility of molecular viability testing by ratiometric pre-rRNA analysis.

In practical terms, pathogen viability testing can be conducted in a clinical sample by dividing the sample into two aliquots, one of which is nutritionally stimulated while the other is held as a non-stimulated control. After brief stimulation, nucleic acid is extracted from both aliquots and subjected to RT-qPCR to quantify species-specific pre-rRNA. When the pre-rRNA increases in the stimulated sample relative to the control sample, the presence of viable cells of the targeted species is indicated. As shown previously [18], the magnitude of pre-rRNA upshift in viable cells is sufficient to enable their detection even when they are greatly outnumbered by inactivated cells. A potential confounding factor is that cells in stimulated and non-stimulated samples may differ with regard to pre-rRNA release upon lytic treatment. For example, stimulated cells might have weaker cell envelopes that release nucleic acid more readily than non-stimulated cells. Additionally, inhibitors present in serum might reduce the efficiency of pre-rRNA amplification in non-stimulated samples relative to stimulated samples. Either factor could create the appearance of increased pre-rRNA in the stimulated aliquot, even if no new pre-rRNA was produced. In the present study, these factors were controlled by normalizing pre-rRNA to genomic DNA, as in Figures 2, 3, 4, S1, and S2. However, this control adds cost and complexity to the procedure, and in at least some cases it may not be needed, as seen in Figures 5 and S3.

The nutritional stimulation step in pre-rRNA analysis (1–4 hours depending on the targeted species), and the requirement for two measurements to obtain ratiometric results, add complexity to the overall procedure. In samples for which the mere presence of pathogen nucleic acid is often a satisfactory indication of disease (for example, *M. tuberculosis* DNA in sputum), this added complexity would be disadvantageous. But for diagnostic indications that require differentiation of viable and dead cells (e.g. antibacterial treatment monitoring), then the speed, sensitivity, and specificity of pre-rRNA analysis may offer advantages. With additional development and automation, it may be possible to generate results faster and with less hands-on work.

The results in Figure 2, although generated with simulated samples, illustrate the potential clinical value of pre-rRNA analysis. Viewed in isolation, the genomic DNA signals in Figure 2 would have suggested dense infections with *P. aeruginosa* and *A. baumannii,* and somewhat lower-grade infection with *S. aureus*. However, the *P. aeruginosa* cells were inactivated while the *S. aureus* cells were partially viable. Therefore, the latter might present a more serious threat to a patient if seen in a real sample. Ratiometric pre-rRNA analysis was able to make this distinction.

## Supporting Information

Figure S1 **Ratiometric pre-rRNA analysis of** ***A. baumannii*** **,** ***S. aureus*** **, and** ***P. aeruginosa*** **cells in serum.** Cells that had been held in serum for 7 days were analyzed as in Figure 2. Viable cell densities of *A. baumannii, S. aureus,* and *P. aeruginosa,* respectively, in serum were 2.94×10^9^, 4.0×10^4^, and <1×10^2^ CFU/mL. From separate gDNA standard curves consisting of ≥ six points each, qPCR efficiencies were calculated to be between 1.030 and 1.077.
(TIF)

Figure S2 **Ratiometric pre-rRNA analysis** ***M. tuberculosis*** **H37Ra cells in serum.** Cells (4.5E7 CFU/mL) were incubated in human serum at 37°C for 30 days. The serum-incubated cells were then resuspended in pre-warmed 7H9 broth and samples were taken after 0,1, 2, 4, and 24 hours later. Pre-rRNA normalized to genomic DNA (P:G) was determined as in Figure 4, except that DNA and RNA were extracting by using the Qiagen Allprep kit. This resulted in relatively poor RNA recovery and thus lower P:G ratios, however rapid upshift of these values were seen after nutritional stimulation, as in Figure 4. From a five-point gDNA standard curve, qPCR efficiency was calculated to be 0.973.
(TIF)

Figure S3 **Ratiometric pre-rRNA analysis of** ***A. baumannii*** **cells in serum by using a rapid semi-automated approach.** Serum-incubated cells were plated to quantify viable CFU/mL, serially diluted in serum, then nutritionally stimulated as in Figure 5. Pre-rRNA was quantified by the rapid proticol used in Figure 5. Values are means and SDs of ΔCt values (non-stimulated minus stimulated) from two replicates of each dilution. Control samples with no bacteria (0 CFU/mL) yielded no RT-qPCR results, and therefore could not be plotted as ΔCt values. Reaction efficiency could not be calculated for this experiment, because no standard curve was used.
(TIF)
